# Adaptive Treatment for Youth with Co-occurring Substance Use and Depression: Long-term Outcome

**DOI:** 10.1192/j.eurpsy.2026.10447

**Published:** 2026-07-31

**Authors:** Y. Kaminer

**Affiliations:** Psychiatry, University of Connecticut, Farmington Connecticut, United States

## Abstract

**Introduction:**

Co-occurring youth substance use and depression are common. The literature on treatment of this dual diagnosis that increase the likelihood of suicidal behavior is meager.

**Objectives:**

To investigate the long-term outcomes among youth with substance use and depression, who enrolled in the Treatment for Teens with Alcohol Abuse and Depression (T-TAAD, Curry J., Kaminer Y., et al. JAACAP, 2022) study. First, identify predictors for end of treatment outcomes. Second, compare trajectories of outcomes from baseline to 9 months post end-of- treatment (EOT) across study arms: early depression responders (EDR), and non-EDR randomized into either cognitive behavioral therapy or enhanced treatment as usual.

**Methods:**

All treatment participants (n=95) aged 14-21 years (Mean 17.4; SD=1.8) were invited to participate in three quarterly post EOT of a 14-week treatment period. Multiple imputation logistic regression models were used to identify predictors of three outcomes: depression remission, alcohol and cannabis abstinence. The long-term trajectory covers seven assessment timepoints: baseline, four, nine, and 14 weeks. Also, three, six-, and nine-months post EOT. Mixed-effect and generalized mixed-effect models were used to examine the trajectories of the following outcomes: The Children’s Depression Rating Scale (CDRS-R) total score, alcohol use and heavy use frequencies, cannabis using days, cannabis urine test, the Children’s Global Assessment Scale (CGAS) score, and Suicide Ideation Questionnaire-Junior (SIQ-JR) score.

**Results:**

All treatment participants (n=95) aged 14-21 years (Mean 17.4; SD=1.8) were invited to participate in three quarterly post EOT of a 14-week treatment period. Multiple imputation logistic regression models were used to identify predictors of three outcomes: depression remission, alcohol and cannabis abstinence. The long-term trajectory covers seven assessment timepoints: baseline, four, nine, and 14 weeks. Also, three, six-, and nine-months post EOT. Mixed-effect and generalized mixed-effect models were used to examine the trajectories of the following outcomes: The Children’s Depression Rating Scale (CDRS-R) total score, alcohol use and heavy use frequencies, cannabis using days, cannabis urine test, the Children’s Global Assessment Scale (CGAS) score, and Suicide Ideation Questionnaire-Junior (SIQ-JR) score.

**Conclusions:**

Youth who responded early (EDR), had better substance use, depression and functioning long-term outcomes than the non-EDR.

**Disclosure of Interest:**

None Declared

